# Olfactory receptor neurons express olfactory marker protein but not calpain 5 from the same genomic locus

**DOI:** 10.1186/s13041-019-0474-z

**Published:** 2019-06-04

**Authors:** Noriyuki Nakashima, Kie Nakashima, Akiko Takaku-Nakashima, Makoto Takano

**Affiliations:** 1 Department of Physiology, Kurume University School of Medicine, 67 Asahi-machi, Kurume-shi, Fukuoka, 830-0011 Japan; 2 Laboratory of Developmental Neurobiology, Graduate School of Biostudies, Kyoto University, Yoshida Hon-machi, Kyoto, 606-8501 Japan

**Keywords:** Olfactory marker protein, Calpain 5, Olfactory receptor neurons, Nested gene, Maturation

## Abstract

Gene expression is highly regulated to functionally diversify cells. Genes that cooperate in the same physiological processes occasionally reside within nearby regions in a chromosome. Olfactory marker protein (OMP) is highly expressed in mature olfactory receptor neurons (ORNs), but its physiological roles are not fully understood. According to the genomic map, the OMP gene is located within an intron of the calcium-dependent protease, calpain 5 (CAPN5); in other words, the OMP gene is a nested intronic gene. Thus, we attempted to investigate the gene expression and protein distribution of CAPN5 in the olfactory epithelium compared with that in the central nervous system (CNS). By performing reverse-transcriptase PCR and in situ hybridization, we confirmed that CAPN5 mRNA was expressed in the olfactory epithelium. We then performed immunohistological investigations using sliced preparations obtained from mice expressing GFP under OMP promoter activity. The detected GFP fluorescence was restricted to the knob, soma and axon bundles of the ORNs, while CAPN5 immunoreactivity (CAPN5-IR) was ubiquitously detected in the olfactory epithelial layer and lamina propria; signals were strongly detected in the supporting cells within the epithelium. In the CNS, CAPN5 signals were widely detected and were especially strong in the hippocampal formation and the piriform cortex as previously indicated. Therefore, these data indicate that ORNs express OMP but not CAPN5 from CAPN5 gene expression even though they are localized in the same genomic locus. The mechanisms by which the OMP promoter is regulated require detailed investigations.

**Electronic supplementary material:**

The online version of this article (10.1186/s13041-019-0474-z) contains supplementary material, which is available to authorized users.

## Introduction

Tens of thousands of genes have been identified, including in humans [1, 2], and the regulation of gene expression attracts scientists’ interest, particularly how gene expression profiles are involved in pathogenesis [3]. Many genes are selectively expressed in certain populations of cells at certain stages and are utilized as molecular markers for stem cells, mature cells and even cancer cells, sometimes without any indication of their physiological functions [4–12]. Olfactory marker protein (OMP) is highly expressed in mature olfactory receptor neurons (ORNs) and has been used as a powerful marker to investigate the physiology of olfaction [11, 12]. Despite its involvement in olfaction [13, 14], the physiological roles of OMP remain unclear. The OMP gene is incorporated within the gene corresponding to a member of the calcium-dependent cysteine protease superfamily, calpain 5 (CAPN5); namely, OMP is a nested gene. This gene structure is conserved in the *Fugu*, rat and human genomes [15]. However, the differential expression profiles of OMP and CAPN5 remain unexplored. Calpains sense changes in intracellular Ca^2+^ levels and regulate cell fate, such as neuronal death and phagocytosis. In olfaction, cAMP and Ca^2+^ operate in concert to transduce olfactory information in the cilia [16], where OMP reportedly regulates Ca^2+^ extrusion via Na^+^-Ca^2+^ exchangers [17] or modulates cAMP-gated Ca^2+^-permeable channels, resulting in reduced Ca^2+^ influx [18, 19]. Thus, OMP may negatively cooperate with calpains. Although co-operational genes often reside in the same chromosome, such as inwardly rectifying potassium channel 6 and sulfonyl urea receptor genes [20, 21], the cooperative expression of nested genes is not completely understood [22, 23]. Thus, we investigated whether ORNs and other neural cells express CAPN5 together with OMP.

## Results

### Calpain 5 was expressed in the olfactory epithelium

**Fig. 1:**
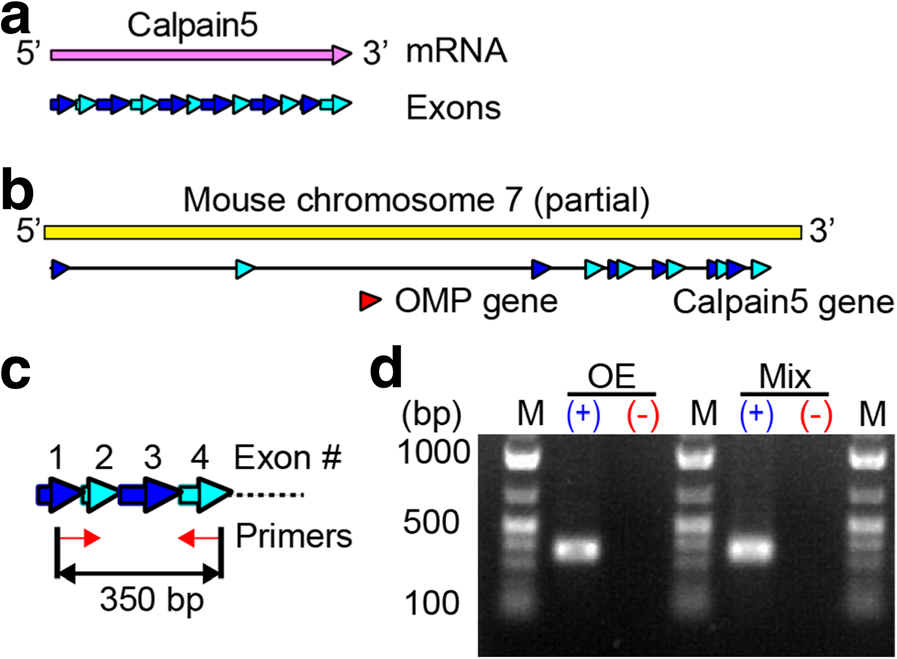
Calpain 5 was detected in the olfactory epithelium. (**a**) The structure of CAPN5 mRNA. (**b**) The coding exons of OMP and CAPN5 on mouse chromosome 7. (**c**) The strategy to amplify CAPN5 cDNA. (**d**) PCR products using cDNAs from OE and whole-brain mixture (Mix) in the presence (+) or absence (−) of reverse transcriptase. The amplicon size was 350 base pairs (bp). M, DNA size marker

CAPN5 mRNA consists of 12 coding exons (Fig. 1a). The OMP gene consists of a single exon and is incorporated between exons 2 and 3 of the CAPN5 gene in the same orientation and on the same strand of chromosome 7 in the mouse (Fig. 1b). We obtained cDNAs from the olfactory epithelium and the brain and examined the expression profiles of CAPN5 mRNA. The primers designed were specific to CAPN5 mRNA but not the CAPN5 gene in the genome; the forward primer annealed to the junction of exons 1/2, while the reverse primer annealed in exon 4 to sandwich the skipped intron region containing the OMP gene (Fig. 1c). We confirmed the expression of CAPN5 mRNA in the olfactory epithelium and the brain (Fig. 1d).

### Calpain 5 immunoreactivity was widely detected

**Fig. 2:**
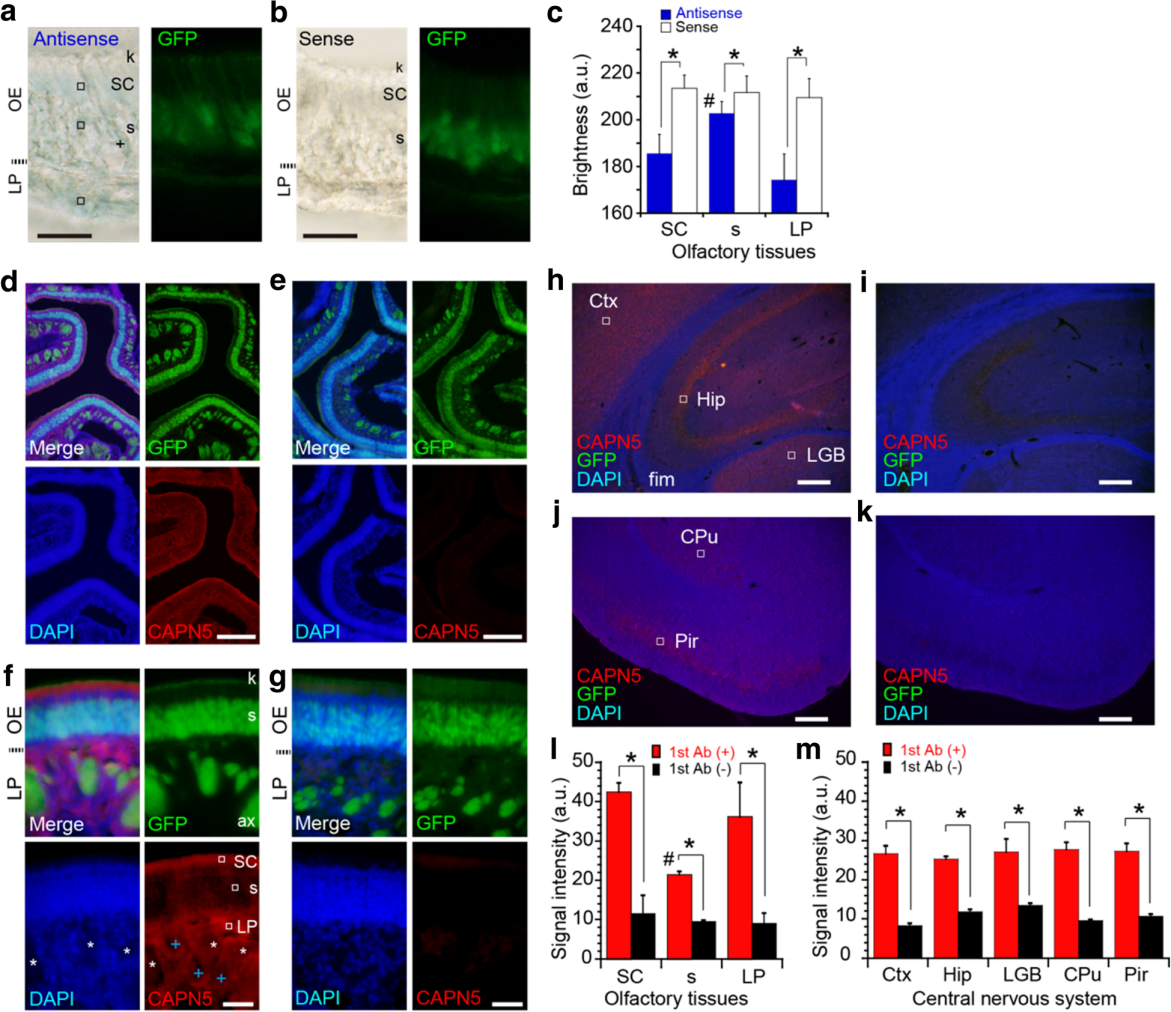
The localization of calpain 5 in the olfactory epithelium. **a, b**, CAPN5 mRNA in situ hybridization images (left panels, bright field) in the presence of an antisense (**a**) or sense (**b**) probe and GFP images (right panels, green; **a**, **b**) in the same areas. Note that the strong signals were detected along the duct from Bowman’s gland (indicated by +). k and s indicate the knob and soma of ORNs, respectively. **c**, Brightness values that declined in the presence of dye in (**a**, **b**) are defined as signals are and shown as 8-bit brightness values in an arbitrary unit (a.u.; 2^8^ as the max). Statistics; * *P* < 0.001; the T-values (df) are 11.7 (38), 4.61 (38), and 11.0 (38) for SC, s, and LP (blue versus white), respectively. ROIs (20 total). Signals were compared between soma and the other olfactory regions; # P < 0.001; the T-values (df) are 7.73 (38) and 9.21 (38) for SC and LP, respectively. **d**-**g,** Immunohistochemistry images (red) with nuclear staining with 4′,6-diamidino-2-phenylindole (DAPI, blue) and GFP fluorescence (green) at low magnification (**d**, **e**) and at high magnification (**f**, **g**) in the olfactory regions in the presence (**d**, **f**) or absence (**e**, **g**) of the anti-CAPN5 primary antibody (1st Ab). **h**-**k**, Verification of CAPN5 antibodies in the other brain regions in the presence (**h**, **j**) or absence (**i**, **k**) of 1st Ab. CAPN5-IR (red), DAPI (blue) and GFP fluorescence (green). fim, fimbria. The GFP signals represent the OMP-expressing ORNs and projections. Only the merged images are presented in (**h**) to (**k**). **l**, **m,** The CAPN5-IR signal intensities (a.u.; 2^8^ as max) for olfactory tissues (**l**) and the central nervous system (**m**). Statistics; * P < 0.001; the T-values (df) are 31.9 (100), 60.2 (114), 18.4 (115), 34.9 (99), 97.1 (99), 18.3 (98), 32.4 (105) and 37.8 (114) for SC, s, LP, Ctx, Hip, LGB, CPu and Pir (red versus black), respectively. To compare CAPN5-IRs between soma and other tissue regions, the same ROIs were statistically analyzed; # P < 0.001; the T-values (df) are 31.1 (112), 10.8 (124), 14.2 (112), 26.9 (111), 9.8 (111), 17.8 (115) and 17.8 (124) for s in (**f**) against SC, LP, Ctx, Hip, LGB, CPu and Pir (red versus red), respectively. ROIs (50–63 total) of the experiments with or without the 1st antibody. Representative ROIs are shown in open rectangles: 30 μm^2^ in (**a, f**); otherwise, 1400 μm^2^ in (**h**) and (**j**). Scales: 30 μm in (**a**, **b**, **f**, **g**); otherwise, 200 μm. Dotted lines in (**a**, **b**, **f**, **g**) indicate the basal membrane separating OE and LP

We next performed immunohistochemistry to localize the protein translation of CAPN5 using *OMP*^*GFP/GFP*^-knockout mice, where OMP-expressing cells were visualized by GPF fluorescence [12]. In situ hybridization revealed that CAPN5 mRNA is widely expressed in the olfactory epithelium and the lamina propria (OE and LP, respectively, Fig. 2a and b). The deposition of dye resulted in a decline in signals intensities; the signals were significantly weaker in the soma (s) of ORNs than those in the supporting cells (SC) and the cells in the LP (Fig. 2c). The immunoreactivity (IR) of CAPN5 was ubiquitously detected in the LP, but only slight signals were detected in the olfactory epithelial layer (OE; Fig. 2d-g). The validity of the antibody for CAPN5 was confirmed in the sensory cortex (Ctx), hippocampus (Hip), lateral geniculate body (LGB), caudate/putamen (CPu) and piriform cortex (Pir) (Fig. 2h-k). According to the mRNA expression database [24], the in situ hybridization results for CAPN5 mRNA were consistent with our results for CAPN5 immunoreactivity (CAPN5-IR).

Upon close observation of the epithelial layer, CAPN5-IR was found mostly in the SC on the apical surface. CAPN5-IR was undetected in the knob layer of ORNs (k; Fig. 2f and g), and the apical signals were confined within the SC. Although weak reticular signals were observed in the somatic layer of ORNs (s; Fig. 2e and f), the cells in the LP uniformly presented CAPN5-IR (Fig. 2f and g). The organs void of CAPN5-IR were morphologically identified as the axon bundles (ax) and the Bowman glands (white asterisks and blue crosses, respectively; Fig. 2f). The GFP signals in the knobs and axons of the ORNs overlapped only slightly with CAPN5-IR (Fig. 2f and g). The reticular distribution of CAPN5-IR weakly encircling the nuclei represents the cytoplasm of the ORNs and the intervening projections of the SC (Fig. 2e). The CAPN5-IR intensity was significantly higher than that in the control samples (Fig. 2l and m).

Taken together, the CAPN5 gene domain is widely active in the olfactory epithelium and central nervous system (CNS), whereas ORNs with strong expression of the nested OMP gene only slightly express the host CAPN5 gene from the same chromosome.

## Discussion

This is the first observation of the expression profiles of CAPN5 and OMP in ORNs, which seem to be negatively correlated, although both are expressed widely in neural tissues [10, 25, 26]. The expression levels of OMP seem differ across OMP-expressing cells, for example, in the hypothalamus, where CAPN5-IR was also detected (Additional file 1: Figure S1a-d). How the CAPN5 genomic locus is regulated to express CAPN5 and/or OMP generally remains unknown. Intron 2 of CAPN5 is considerably long (16 kb) compared to the entire CAPN5 gene (60 kb). Phylogenetically, while a CAPN5 ortholog is found in *Caenorhabditis elegans* (*C. elegans*) [27], according to an NCBI BLAST search, the *C. elegans* genome contains no OMP ortholog. Thus, the OMP gene is expressed independently from CAPN5. Notably, the OMP gene in vertebrates lacks canonical TATA and CAAT boxes [28], but the transcription factor Olf-1 binds less than 0.3 kb upstream of the OMP gene and can direct OMP expression selectively in ORNs [29, 30]. Olf-1, together with early B-cell factor (O/E), forms an O/E family consisting of four homologs, all of which are expressed in the ORN layer at different levels, suggesting that O/E members may operate as substitutes or in combination at various maturation stages [31, 32]. Surprisingly, mutation at this binding site does not affect the spatiotemporal expression patterns of OMP in ORNs [33]. Notably, growth-associated protein 43 (GAP43) and OMP are expressed in a reciprocal fashion during maturation and regeneration [34, 35]. GAP43 contains several palindromic sequences resembling Olf-1-binding sites within introns (Additional file 1: Figure S2a and b) [29, 36]. Binding of O/E family members within introns may affect the transcription or splicing of host genes. Olf-1-binding sites have also been identified in ORN-related genes, including adenylate cyclase 3 (AC3) and Gα_olf_ [29, 32]. How Olf-1 operates is unclear, and manipulating the OMP locus may require simultaneous attention to CAPN5 expression. Moreover, Gα_olf_ is also expressed in the striatum [37], and AC3 has recently been considered a marker of sensory and primary cilia [38]. Primary cilia are widely found in many cells, including neural cells and blood cells, operating as cellular antennae for development via Sonic hedgehog (Shh) signaling [39–43]. Shh in the olfactory mucus and epithelium is involved in axon growth, ciliary extension and functional localization of AC3 and odorant receptors in the cilia, though the loss of Shh is irrelevant to OMP expression [44–47]; OMP is initially expressed before the functional maturation of ORNs. Unlike other terminally differentiated neurons, ORNs periodically undergo turnover [48], and OMP remains in the ORNs, even during the apoptotic stage [49]. The primary cilia transform dynamically during neural differentiation [50], and ORNs expressing OMP can be considered functional but intermediate before apoptosis. OMP expression is downregulated in several pathological situations [34, 51], and the gene regulation of OMP is of pathophysiological interest. Lastly, the coordinated expression of multiple genes is sometimes achieved by distant intra- or even inter-chromosomal interactions [52]. Further investigations from these wide viewpoints are certainly needed.

## Methods and materials

### Treatment of animals

We treated experimental animals in accordance with the Kurume University guidelines. Mice expressing GFP under the OMP promoter were obtained from Riken Bioresource Center (RBRC 02092) under permission from Professor Peter Mombaerts (Max Planck, Germany). The animals used were anesthetized by intraperitoneal injection of dexmedetomidine, midazolam and butorphanol (4, 10 and 0.5, in mg/kg, respectively) before rapid decapitation with sharp blades for subsequent experiments.

### Genomic search

We consulted the websites of the National Center for Bioinformatics (NIH, USA) to obtain the relevant gene information.

### Reverse-transcriptase PCR

Olfactory epithelial tissues were collected from the nasal cavities of 8-week-old male mice using cotton swabs and were dissolved in the appropriate solution to extract mRNAs using a kit (Roche, Basel, Switzerland). cDNA was synthesized from mRNA using Superscript™ IV (Invitrogen, CA, USA). cDNA synthesized from whole-brain total RNA was used as a positive control. The forward primer for CAPN5 (NM_007602) was 5′-CGGCCTAAGGATATCTGCGACGATC-3′; the reverse primer was 5′-TTGGCATAGGCCTTCTCCACCAG-3′. The samples were subjected to 35 cycles of 98 °C for 10 s, 52 °C for 20 s, and 72 °C for 30 s. The PCR product was used for in situ hybridization.

### Fluorescent immunohistochemistry

Three mice were perfusion-fixed with 4% paraformaldehyde in phosphate-buffered saline (PBS). Then, the skull was post-fixed for 10 h, decalcified in PBS containing 250 mM EDTA for 7 days, cryo-protected by overnight incubation in PBS containing 30% w/v sucrose, mounted in OCT Embedding Compound (Sakura Finetek, Tokyo, Japan) and frontally sectioned at 25-μm thickness using a cryostat (CM3050S, Leica Microsystems, Wetzlar, Germany). Sections were then incubated at room temperature overnight in an appropriate blocking solution containing an anti-CAPN5 primary antibody (ab28280, Abcam, CA, USA), washed with PBS containing 0.3% Triton X-100 (PBS-X), incubated with DAPI and the appropriate secondary antibody (Alexa-Fluor 594 conjugated, 1:200; Molecular Probes, USA; in blocking solution) for 1.5–2 h, washed in PBS-X, mounted onto MAS-coated glass slides (Matsunami Glass, Osaka, JAPAN), coverslipped using Vectashield antifade reagent (Vector Labs, CA, USA) and tightly sealed. The fluorescence signals were detected using a fluorescent microscope (BX50; Olympus, Tokyo Japan) and were analyzed with cellSens image analysis software (Olympus, Tokyo, Japan). The images for CAPN5-IR were captured by exposing the samples for 40 ms at low magnification (× 10, objective) and for 80 ms at high magnification (× 60) and were analyzed offline with NIH ImageJ (MD, USA).

### In situ hybridization for CAPN5 mRNA

The PCR product was phosphorylated using T4 polynucleotide kinase (Takara Bio Inc., Shiga, Japan) and inserted into the EcoRV site of the pBluescript II KS (+) plasmid (Agilent Technologies, CA, USA). Antisense and sense probes for detecting CAPN5 mRNA were generated from the plasmid by T3 and T7 RNA polymerases (Sigma-Aldrich, MO, USA). The animals were fixed by cardiac perfusion of 4% paraformaldehyde in PBS. The olfactory epithelium and brain were sliced at (20 μm). The samples were incubated for 5 min at 37 °C with 10 μg/ml proteinase K (Wako Pure Chemical, Osaka, Japan) in 0.1 M Tris, pH 8.0, and 50 mM EDTA, acetylated for 10 min with 0.25% acetic anhydride in 0.1 M triethanolamine, washed two times for 5 min each with 2 × Standard Saline Citrate (SSC) at room temperature, pre-incubated for 2 h at room temperature in a prehybridization solution containing 50% deionized formamide, 5 × SSC, 5 × Denhardt’s solution (Wako Pure Chemical, Osaka, Japan), 250 μg/ml yeast tRNA (Sigma-Aldrich, MO, USA), and 500 μg/ml salmon sperm DNA (Thermo Fisher Scientific, MA, USA), and then incubated at 55 °C overnight in prehybridization solution containing the denatured Dig-labeled probes (1.25 μg/ml, Sigma-Aldrich, MO, USA). The samples were then washed three times for 20 min each with 4 × SSC, and nondigested probes were degraded by 30-min incubation at 37 °C in RNase solution containing 20 μg/ml RNase A (Sigma-Aldrich, MO, USA), 500 mM NaCl, 0.1 M Tris, pH 8.0, and 1 mM EDTA. The samples were then washed with serially diluted SSC solutions containing 1 mM dithiothreitol (2 × SSC for 5 min two times, 1 × SSC for 5 min, 0.5 × SSC for 5 min, 0.1 × SSC for 30 min at 65 °C, and 0.1 × SSC for 5 min). The samples were then incubated in prehybridization solution with 1:2000-diluted anti-Dig-antibodies conjugated with alkaline phosphatase (Sigma-Aldrich, MO, USA) at 4 °C overnight. The samples were washed and incubated in PBS containing nitro blue tetrazolium chloride and 5-bromo-4-chloro-3-indolyl phosphate salt (Sigma-Aldrich, MO, USA). Sense-RNA was used as a control. The images were captured by exposing the samples for 1 ms and were analyzed offline with NIH ImageJ. Signal intensities were measured as the reduction of brightness.

### Online search for transcription factor binding sites

We analyzed transcription factor binding sites for the CAPN5 and GAP43 genes using an online search engine (Tfsitescan, RRID: SCR_010667) [53]. The upstream sequences between a TATA box and a Kozak sequence were analyzed.

### Statistical analysis

Statistical analysis was performed using the unequal variance unpaired T-test. *P* values less than 0.001 were considered to indicate a significant difference in the means. T-values and degrees of freedom (df; within parentheses) are shown in the legend for Fig. 2.

## Additional file

Additional file 1: **Figure S1.** The localization of CAPN5 in the hypothalamus. **a-d,** CAPN5-immunoreactivities in the presence (**a, b**) and absence (**c, d**) of primary antibody (1st Ab). PH, posterior hypothalamic area. VMH, ventromedial hypothalamus. Mtu, medial tuberal nucleus. cp, cerebral peduncle. 3 V, the third ventricle. Scales: 200 μm. **Figure S2** The mouse CAPN5 and GAP43 genes contain palindromic sequences resembling Olf-1-binding sites within introns. (**a**) The OMP gene, between exons 2/3 of CAPN5, contains an Olf-1-binding site upstream of the start codon but no Kozak sequence or TATA/CAAT boxes. (**b**) The GAP43 gene contains TATA/CAAT boxes and a Kozak sequence, except for palindromic sequences (CCCNNGGG) such as Olf-1-binding sites upstream, near the start codon. The nucleotide N indicates any of A, T, C or G. Most introns are not shown. kb, kilobase pairs. No binding sites for Olf-1 were predicted upstream of GAP43 or CAPN5. The 1st and 2nd introns of GAP43 were predicted to potentially contain binding sites for Olf-1. (DOCX 1012 kb)

## Data Availability

The datasets analyzed for this study are included in the manuscript and the Additional file 1.

## Abbreviations

AC3: Adenylate cyclase 3
CAPN5: Calpain 5
CNS: Central nervous system
CPu: Caudate/putamen
Ctx: Sensory cortex
DAPI: 4′,6-diamidino-2-phenylindole
GAP43: Growth-associated protein 43
Hip: Hippocampus
IR: Immunoreactivity
LGB: Lateral geniculate body
LP: Lamina propria
O/E: Olf-1 and early B-cell factor
OE: Olfactory epithelium
OMP: Olfactory marker protein
ORNs: Olfactory receptor neurons
PBS: Phosphate-buffered saline
Pir: Piriform cortex
s: Soma
SC: Supporting cells
