# Supplementary material for: Olfactory receptor neurons express olfactory marker protein but not calpain 5 from the same genomic locus
Source: Mol Brain. 2019 Jun 4;12:54. doi: 10.1186/s13041-019-0474-z (PMC6549253; doi:10.1186/s13041-019-0474-z)
Supplement: Supplementary file 1 — Figure S1. The localization of CAPN5 in the hypothalamus. a-d, CAPN5-immunoreactivities in the presence (a, b) and absence (c, d) of primary antibody (1st Ab). PH, posterior hypothalamic area. VMH, ventromedial hypothalamus. Mtu, medial tuberal nucleus. cp, cerebral peduncle. 3 V, the third ventricle. Scales: 200 μm. Figure S2 The mouse CAPN5 and GAP43 genes contain palindromic sequences resembling Olf-1-binding sites within introns. (a) The OMP gene, between exons 2/3 of CAPN5, contains an Olf-1-binding site upstream of the start codon but no Kozak sequence or TATA/CAAT boxes. (b) The GAP43 gene contains TATA/CAAT boxes and a Kozak sequence, except for palindromic sequences (CCCNNGGG) such as Olf-1-binding sites upstream, near the start codon. The nucleotide N indicates any of A, T, C or G. Most introns are not shown. kb, kilobase pairs. No binding sites for Olf-1 were predicted upstream of GAP43 or CAPN5. The 1st and 2nd introns of GAP43 were predicted to potentially contain binding sites for Olf-1. (DOCX 1012 kb) [file 13041_2019_474_MOESM1_ESM.docx]

**Additional file 1:**

**Olfactory receptor neurons express olfactory marker protein but not calpain 5 from the same genomic locus**

Noriyuki Nakashima, Kie Nakashima, Akiko Takaku-Nakashima and Makoto Takano


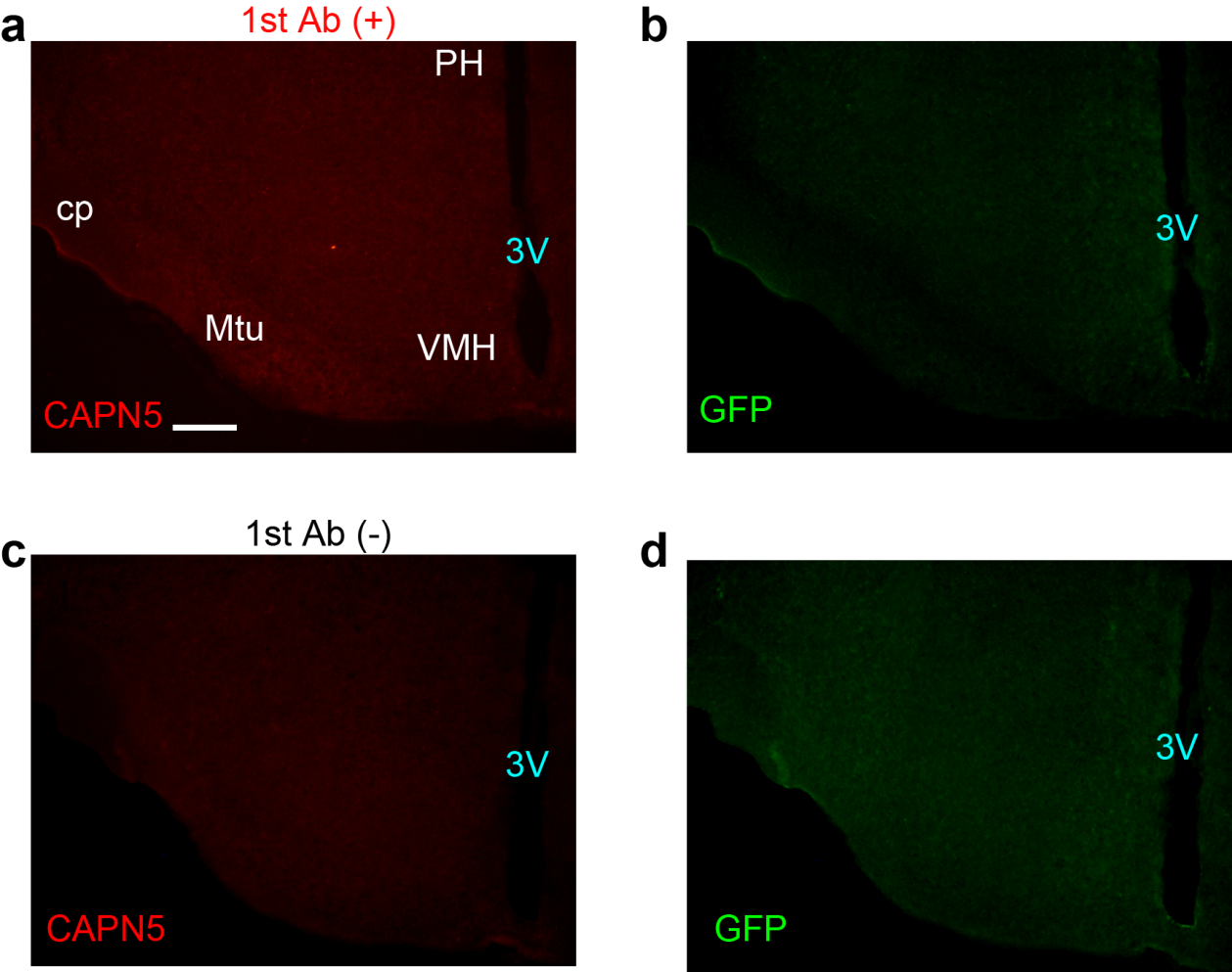


**Figure S1.** The localization of CAPN5 in the hypothalamus. **a-d,** CAPN5-immunoreactivities in the presence (**a, b**) and absence (**c, d**) of primary antibody (1st Ab). PH, posterior hypothalamic area. VMH, ventromedial hypothalamus. Mtu, medial tuberal nucleus. cp, cerebral peduncle. 3V, the third ventricle. Scales: 200 μm.

**
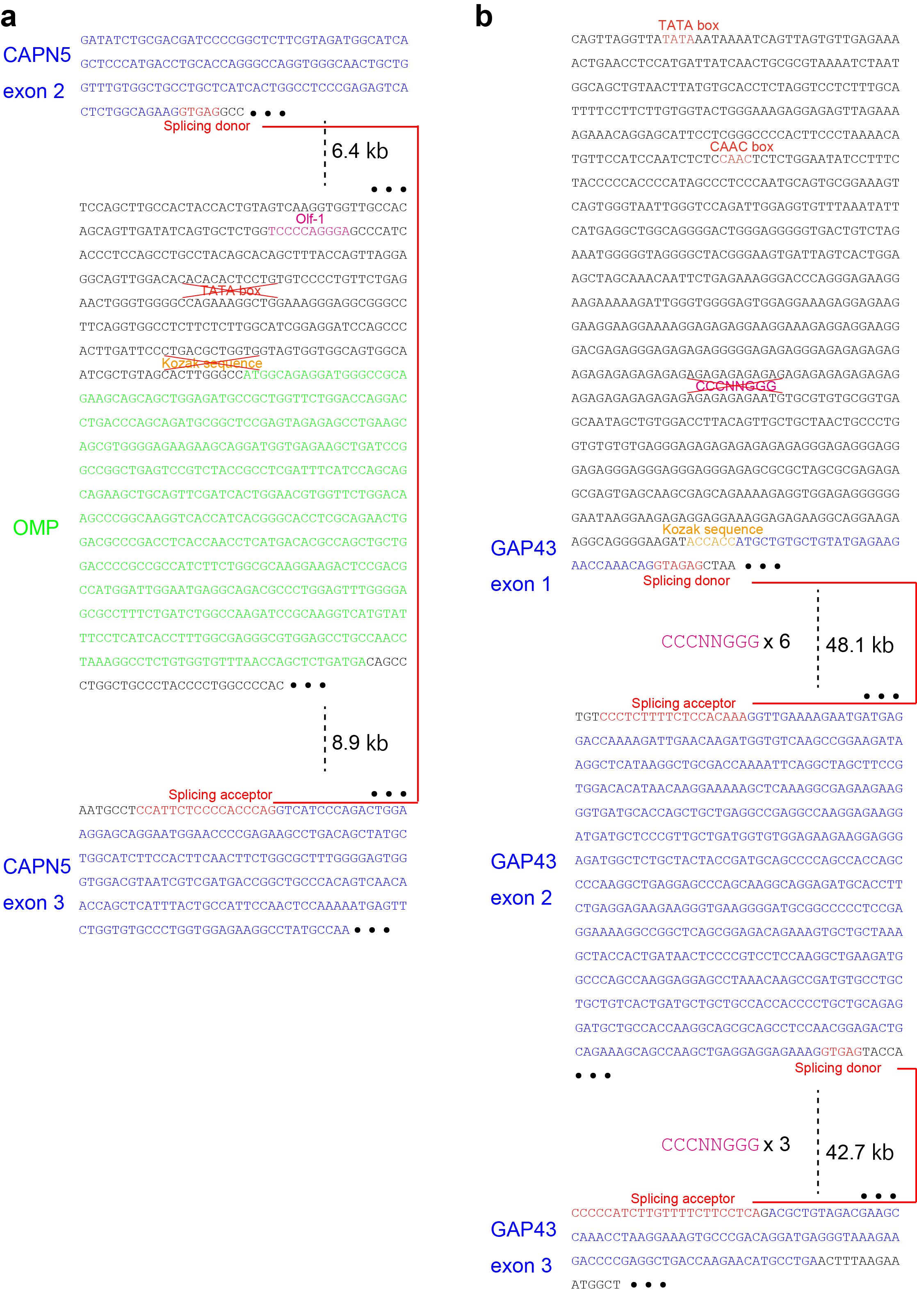
**

**Figure S2.** The mouse CAPN5 and GAP43 genes contain palindromic sequences resembling Olf-1-binding sites within introns. (**a**) The OMP gene, between exons 2/3 of CAPN5, contains an Olf-1-binding site upstream of the start codon but no Kozak sequence or TATA/CAAT boxes. (**b**) The GAP43 gene contains TATA/CAAT boxes and a Kozak sequence, except for palindromic sequences (CCCNNGGG) such as Olf-1-binding sites upstream, near the start codon. The nucleotide N indicates any of A, T, C or G. Most introns are not shown. kb, kilobase pairs. No binding sites for Olf-1 were predicted upstream of GAP43 or CAPN5. The 1^st^ and 2^nd^ introns of GAP43 were predicted to potentially contain binding sites for Olf-1.
